# Influence of Teaching Efficacy and Competence on Life Satisfaction in Pre-Service Physical Education Teachers: Is There a Gender Difference?

**DOI:** 10.3390/healthcare13162055

**Published:** 2025-08-20

**Authors:** Ginés David López-García, María Carrasco-Poyatos, Rut López-Osca, Antonio Granero-Gallegos

**Affiliations:** 1 Department of Plastic, Musical and Dynamic Expression, University of Murcia, 30100 Murcia, Spain; 2 Department of Education, University of Almeria, 04120 Almeria, Spain; 3 Health Research Centre, University of Almeria, 04120 Almeria, Spain

**Keywords:** subjective well-being, psychological needs, pre-service teacher training, gender differences, SDT, motivation

## Abstract

**Purpose**: Grounded in Social Cognitive Theory and Self-Determination Theory, this study analyzed gender differences in the relationships between teachers’ sense of efficacy, basic psychological need satisfaction and frustration (competence), and life satisfaction among Physical Education (PE) pre-service teachers. **Method**: A sample of 368 PE pre-service teachers (Mage = 23.41 ± 2.37; 48.1% women) participated. A multi-group structural equation modeling approach was used. **Results**: Male participants reported significantly higher levels of competence satisfaction compared to their female counterparts. Teaching efficacy positively predicted life satisfaction, both directly and indirectly via competence satisfaction. Notably, the indirect effects were stronger among women, while direct effects were observed only in the female group. **Conclusions**: The findings emphasize the key role of competence satisfaction in explaining how teaching efficacy influences life satisfaction in pre-service teachers. Gender differences suggest that while both men and women benefit from feeling competent, the pathways differ, highlighting the importance of gender sensitive strategies in teacher education programs.

## 1. Introduction

Psychological well-being is widely recognized as a critical component within the teaching profession [1]. In the context of teacher education, particular emphasis has been placed on life satisfaction, given its relevance as a key predictor of success in the teaching career [1,2]. Throughout the training process, pre-service Physical Education (PE) teachers who report higher levels of life satisfaction are more likely to develop the essential skills and competencies required for effective professional practice [3]. Consequently, exploring the levels of life satisfaction among pre-service teachers is of great importance, especially considering their role as future role models within educational environments [4,5]. Drawing on Social Cognitive Theory (SCT) [6], the concept of teachers’ sense of efficacy emerges as a key cognitive factor in teacher preparation, as it has the potential to shape teachers’ experiences of well-being [7]. While previous research has primarily focused on the relationship between various factors and psychological well-being (i.e., life satisfaction) in preservice teachers [8,9,10], little attention has been paid to how teaching efficacy during initial teacher education may influence life satisfaction in pre-service PE teachers.

Studies in teacher training have consistently highlighted gender differences in cognitive factors like teaching efficacy [3,11]. Specifically, research suggests that female pre-service teachers tend to report higher levels of teaching efficacy than their male colleagues during their training process [3]. Therefore, future studies may benefit from adopting a gender-sensitive perspective to help clarify the relationship between teachers’ sense of efficacy and life satisfaction among PE pre-service teachers. The limited number of studies in this area highlights the need to consider gender as a relevant variable when analyzing the impact of teaching efficacy on broader indicators of well-being, such as life satisfaction. In this regard, analyzing the interplay between these variables from a gender-informed perspective allows for a deeper understanding of how psychological mechanisms, particularly those proposed by Self-Determination Theory (SDT) [12], operate throughout teacher training. Specifically, identifying the influence of psychological mediators, such as the need for competence, may provide insights into how pre-service teachers’ perceptions of their future teaching roles are shaped by their experience of competence, whether satisfied or frustrated, during their preparation. Following the SCT framework [6], the present study seeks to extend previous evidence by examining gender differences in the relationship between teachers’ sense of efficacy and life satisfaction in pre-service PE teachers.

### 1.1. Teacher’s Sense of Efficacy

Social Cognitive Theory posits that human beings are capable of acting as causal agents in shaping the course of their own lives [6]. According to Bandura [6], individuals who view themselves as efficacious are more likely to adopt higher goals and sustain greater psychological investment in the pursuit of those goals. Within the teacher education context, teacher self-efficacy is defined as pre-service teachers’ beliefs in their capability to organize and execute the actions required for effective teaching [6]. Accordingly, pre-service teachers who feel efficacious are more likely to set higher goals, persist in challenging tasks, and invest more effort in their training, which contributes to their well-being and personal development [6]. In line with the theoretical underpinnings of SCT, prior research has shown that teaching efficacy plays a critical role in shaping the cognitive and motivational processes of pre-service teachers during their initial training [6,7,13]. Indeed, research focused on pre-service teachers has highlighted the connection between teaching efficacy and motivational variables rooted in SDT [12]. Specifically, perceived self-efficacy has been found to enhance individuals’ perceptions and satisfaction of their basic psychological needs, particularly the need for competence, as outlined by SDT. Authors such as Raven and Kleinert [14] and Kassis et al. [15] have reported positive associations between teaching efficacy and competence satisfaction, as well as negative associations with competence frustration. However, Nguyen et al. [16] emphasize a clear distinction between teaching efficacy and the need for competence, whereas perceived self-efficacy refers to one’s beliefs about achieving future outcomes, the need for competence concerns the present experience of feeling effective or ineffective. Accordingly, the SCT framework has also been used to link teaching efficacy with various dimensions of subjective well-being, including life satisfaction [7].

### 1.2. Psychological Needs

SDT is one of the most widely applied psychological frameworks for understanding motivation in educational contexts, particularly in teacher education [17,18]. Within SDT, the Basic Psychological Needs Theory stands out as a key mini-theory that identifies four fundamental psychological needs (i.e., autonomy, competence, relatedness, and novelty) which are considered essential and universal nutrients for optimal development, growth, and well-being. Among these, the need for competence refers to the innate desire to feel effective and capable when engaging in meaningful activities [18,19]. This need is satisfied when individuals skillfully engage in tasks and seize opportunities to enhance their skills and experiences, whereas it is frustrated when they perceive themselves as ineffective, unsuccessful, or powerless. Supporting this theoretical perspective, previous studies, e.g., Refs. [20,21] have demonstrated that life satisfaction is positively associated with competence satisfaction and negatively associated with competence frustration. However, despite increasing attention to this relationship in teacher education settings [22,23], to the best of our knowledge, no prior research has specifically examined how the satisfaction or frustration of the need for competence among pre-service PE teachers influences their psychological well-being.

### 1.3. Life Satisfaction

Life satisfaction is defined as an individual’s cognitive evaluation of their own characteristics in comparison to their perception of life circumstances [24]. It is shaped through interactions with the environment, leading to an overall positive appraisal of one’s life [25]. Several studies in educational contexts [1,5,26,27] have shown that students with high levels of life satisfaction tend to lead goal-oriented lives, demonstrate greater control over their personal development, and are more receptive to growth and progress. Pre-service teachers who exhibit higher levels of life satisfaction are therefore more likely to actively engage in their learning processes by strengthening personal resources and developing essential professional skills during their initial teacher education [27]. This association suggests that future teachers with greater satisfaction in life are more inclined to invest cognitively and motivationally in their academic development. Elevated life satisfaction fosters broader thought-action repertoires, which in turn enhance individual capabilities and professional readiness. From this perspective, investigating the psychological well-being of pre-service teachers is particularly relevant, given their role as future role models in educational settings.

### 1.4. Gender Differences

Despite growing interest, empirical evidence in teacher education has yet to reach consensus on gender differences in key psychological variables such as teaching efficacy, competence satisfaction, and life satisfaction. For instance, some studies have reported no significant gender differences in teachers’ sense of efficacy [28] or in competence satisfaction [29]. In contrast, other studies have found that male students scored higher in teaching efficacy [30], competence frustration [31,32], and life satisfaction [33]. Conversely, some research has shown that female students reported higher levels of teaching efficacy [11], including among PE pre-service teachers [3]. Beyond the existing controversy surrounding gender-based differences, studies specifically addressing the relationship between teaching efficacy and life satisfaction within teacher training contexts remain scarce. Notably, the study by Burgueño et al. [7] was the only one to examine the association between pre-service teachers’ sense of efficacy and life satisfaction. However, this research did not consider the potential mediating role of psychological needs, particularly competence, as proposed by SDT, nor did it explore whether these associations differ between men and women. From a theoretical perspective, examining gender differences in the relationship between teaching efficacy and life satisfaction may provide deeper insights into how perceptions of competence, both its satisfaction and frustration, impact male and female pre-service teachers differently throughout their training. Such an understanding could help identify key factors that foster subjective well-being during the formative stage of teacher education, particularly for those preparing to enter the PE teaching profession.

### 1.5. The Present Study

Given the importance of life satisfaction among pre-service teachers, both during their training and throughout their future professional careers, it is essential to analyze how cognitive and motivational variables contribute to their overall well-being. Moreover, to the best of our knowledge, the mediating role of competence need satisfaction and frustration in the relationship between teaching efficacy and life satisfaction has not yet been thoroughly examined in the existing literature. Based on the reviewed evidence and the theoretical foundations of SCT and SDT, the objective of this study was twofold. First, to examine whether there are significant gender differences among male and female pre-service teachers in cognitive and motivational variables. Second, to analyze the mediating role of academic competence satisfaction and frustration in the relationship between teaching efficacy and life satisfaction. The following hypotheses were established: first, preservice teachers’ sense of efficacy positively predicts life satisfaction (H1); second, competence satisfaction positively mediates the relationship between the preservice teachers’ sense of efficacy and life satisfaction (H2); third, competence frustration negatively mediates the relationship between the preservice teachers’ sense of efficacy and life satisfaction (H3). To guide the reporting of the study, the Strengthening the Reporting of Observational Studies in Epidemiology (STROBE) initiative was followed [34,35].

## 2. Methods

### 2.1. Design and Participants

This research employed an observational, cross-sectional, and descriptive design. The sample consisted of students enrolled in the Master’s in Secondary Education Teaching (MAES) program from various public universities in Andalusia (Spain). The inclusion criteria for participation were (i) enrolment in the MAES program during the 2022/2023 academic year; (ii) regular attendance in face-to-face classes; (iii) provision of informed consent; and (iv) full completion of the questionnaire. An a priori sample-size calculation was conducted using Free Statistics Calculators v4.0 [36] to ensure adequate statistical power for the SEM analyses. For a model with four latent variables and 18 observed indicators, the minimum required sample to achieve 90% power at α = 0.05 for a medium anticipated effect (f^2^ = 0.218) was N = 359. Our achieved sample (N = 369; analytic N = 368) exceeds this requirement, ensuring sufficient power for model estimation and hypothesis testing.

### 2.2. Instrument

#### 2.2.1. Teaching Sense of Efficacy (SEE)

The Spanish version [7] of the Teacher’s Sense of Efficacy Scale by Tschannen-Moran and Hoy [37] was used. This instrument consists of four items assessing instructional strategies (e.g., “To what extent can you craft good questions for your students?”), four items measuring student engagement (e.g., “How much can you do to help students value learning?”), and three items evaluating classroom management (e.g., “How much can you do to control disruptive behavior in the classroom?”). Responses were collected using a 9-point Likert scale ranging from 1 (nothing) to 9 (a great deal). For this study, the scale was treated as a unidimensional factor. CFA indicated a good fit for a one-factor model χ^2^/df = 2.78, *p* < 0.001; CFI = 0.975; TLI = 0.963; RMSEA = 0.070 (90% CI [0.054, 0.085]); SRMR = 0.029, and internal consistency was high (α = 0.92; ω = 0.92).

#### 2.2.2. Competence Need Satisfaction

The Spanish version [38] of the Basic Psychological Needs Satisfaction Scale developed by Gillet et al. [39] was used to assess pre-service teachers’ satisfaction of the need for competence in the context of initial teacher education. The scale is introduced with the phrase “In my classes…” and includes five items specifically related to competence satisfaction (e.g., “I feel I am good at what I do”). Responses were rated on a 5-point Likert scale ranging from 1 (strongly disagree) to 5 (strongly agree). CFA indicated excellent fit for a one-factor model χ^2^/df = 0.99, *p* = 0.423; CFI = 1.000; TLI = 1.000; RMSEA = 0.000 (90% CI [0.000, 0.026]); SRMR = 0.014, and internal consistency was adequate (α = 0.81; ω = 0.82).

#### 2.2.3. Competence Need Frustration

The Spanish version [40] of the Psychological Need Thwarting Scale originally developed by Bartholomew et al. [41] was used to assess pre-service teachers’ frustration with the need for competence. The instrument begins with the phrase “In my classes…” and includes four items focused on competence frustration (e.g., “Situations occur in which I am made to feel incapable”). Items were rated using a 5-point Likert scale ranging from 1 (strongly disagree) to 5 (strongly agree). CFA indicated acceptable fit for a one-factor model χ^2^/df = 1.76, *p* = 0.172; CFI = 0.997; TLI = 0.990; RMSEA = 0.084 (90% CI [0.046, 0.122]); SRMR = 0.015, and internal consistency was adequate (α = 0.80; ω = 0.81).

#### 2.2.4. Life Satisfaction

The Spanish version [42] of the Satisfaction With Life Scale (SWLS) originally developed by Diener et al. [25] was used to assess participants’ overall cognitive judgment of life satisfaction. The instrument begins with the prompt “In relation to my life…” and consists of five items (e.g., “I am satisfied with my life”). Responses were collected using a 7-point Likert scale ranging from 1 (strongly disagree) to 7 (strongly agree). This scale has shown adequate psychometric properties in previous research and was treated as a unidimensional factor in the present study. CFA indicated a good fit for a one-factor model χ^2^/df = 1.34, *p* = 0.253; CFI = 0.998; TLI = 0.995; RMSEA = 0.030 (90% CI [0.000, 0.089]); SRMR = 0.015, and internal consistency was adequate (α = 0.82; ω = 0.82).

### 2.3. Procedure

First, coordinators of the MAES program at each participating university were contacted to request their collaboration. After informing them of the study’s objectives and characteristics, institutional permission was obtained. Students were then contacted via email and invited to complete an online questionnaire, which included details on the study purpose, anonymity of responses, instructions for completing the scales, and the voluntary nature of participation, including the right to withdraw at any time. All participants provided informed consent prior to participation. The study was carried out in accordance with the ethical principles of the Declaration of Helsinki and received approval from the Bioethics Committee of the University of Almería (Ref: UALBIO2021/009).

### 2.4. Data Analysis

All statistical procedures were carried out using SPSS v.29 and AMOS v.27 software. Initially, following Ibáñez-López et al. [43] and Sánchez-Martín et al. [44], descriptive analyses were conducted (i.e., means, standard deviations, and Pearson correlations). Gender-based differences in the study variables were explored through independent samples t-tests. The internal consistency of the measurement instruments was assessed using McDonald’s omega (ω), with values above 0.70 regarded as satisfactory for research purposes [45]. To examine gender-based differences in the relationships among the variables, a multi-group structural equation modeling approach was employed. The model included teaching efficacy, competence satisfaction and frustration, and life satisfaction, and was tested simultaneously for men and women. The university of origin was used as a control variable. Given the violation of multivariate normality in both gender groups (Mardia’s coefficient: men = 7.41, *p* < 0.001; women = 12.44, *p* < 0.001), model estimation was conducted using the maximum likelihood method with 5000 bootstrap resamples. Indirect effects were also examined using bootstrapping and interpreted as statistically significant when the 95% confidence interval did not include zero [46,47]. Model fit was assessed using several indices: chi-square divided by degrees of freedom ratio (χ^2^/df), Comparative Fit Index (CFI), Tucker–Lewis Index (TLI), Root Mean Square Error of Approximation (RMSEA) with its 90% confidence interval, and the Standardized Root Mean Square Residual (SRMR). Acceptable fit thresholds were defined as χ^2^/df < 5.0, CFI and TLI ≥ 0.90, and RMSEA and SRMR ≤ 0.08 [48]. Finally, the coefficient of determination (R^2^) was used to interpret the explained variance in each endogenous variable. Following Domínguez-Lara [49], R^2^ values of 0.02, 0.13, and 0.26 were interpreted as small, medium, and large effect sizes, respectively. Confidence intervals (95%) were reported to confirm their interpretability.

## 3. Results

### 3.1. Participants

A total of 369 students from the MAES program, all specializing in PE and enrolled in public universities of Andalusia, took part in the study. Participants ranged in age from 21 to 61 years (M = 27.28; SD = 6.705). As the study involved gender-based comparisons, one participant who selected “other gender” (0.3%) was excluded from the analyses, resulting in a final sample of 368 students (53.4% male and 46.3% female). The data were collected during May 2022. The dataset contained no missing values.

### 3.2. Preliminary Results

Table 1 presents the descriptive statistics of the study variables by gender. As shown in Table 1, both male and female pre-service teachers exhibited positive associations between teaching efficacy and competence satisfaction. However, only in the male group was teaching efficacy significantly correlated with competence frustration, showing a negative relationship. Notably, the correlation between teaching efficacy and life satisfaction was stronger among male participants, while this association was weaker in the female group.

Table 2 presents the gender mean differences. A statistically significant gender difference was found only in competence satisfaction, with male participants reporting higher mean scores. For teaching efficacy, competence frustration, and life satisfaction, no significant gender-based differences were observed, although mean scores for teaching efficacy were slightly higher among male pre-service teachers.

### 3.3. Main Results

**Figure 1:**
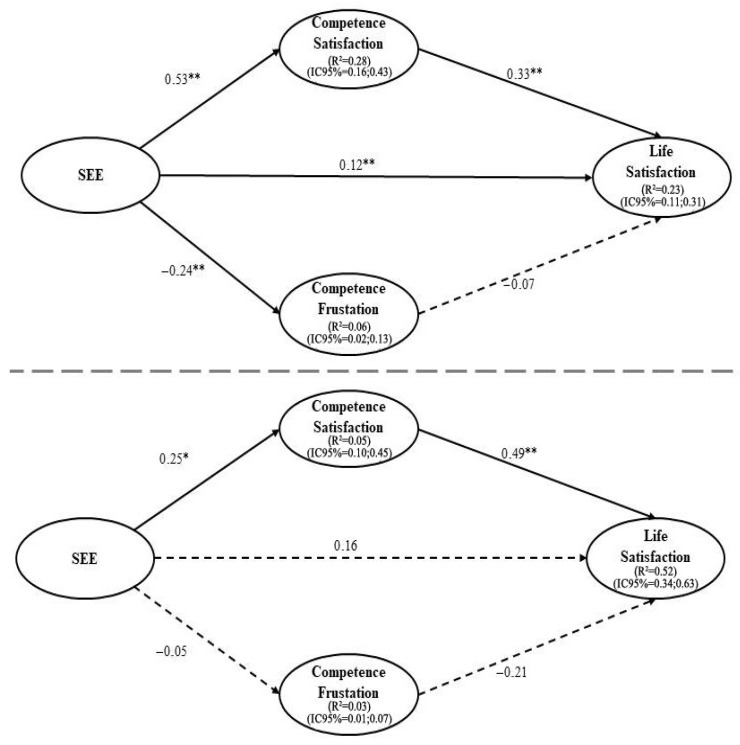
Predictive relationships of the sense of teaching efficacy to Life Satisfaction mediated by competence satisfaction and competence frustration in women (bottom) and men (top). Note: ** *p* < 0.01; * *p* < 0.05. SEE = sense of teaching efficacy; R^2^ = explained variance; CI = confidence interval. The 95% CI is reported in parentheses. The dotted arrows represent the non-significant relationships.

Given the presence of significant gender differences, a multigroup structural equation modeling analysis was performed, separating male and female PE pre-service teachers. In Step 1, the measurement model showed adequate fit to the data: χ^2^/df = 1.814, *p* < 0.001; CFI = 0.936; TLI = 0.922; RMSEA = 0.047 (90% CI = 0.040, 0.054); and SRMR = 0.053. The same goodness-of-fit indices were observed in Step 2: χ^2^/df = 1.814, *p* < 0.001; CFI = 0.936; TLI = 0.922; RMSEA = 0.047 (90% CI = 0.040, 0.054); and SRMR = 0.053. The model was adjusted for participants’ home university and age. Regarding explained variance, the model accounted for 28% of competence satisfaction, 6.1% of competence frustration, and 23% of life satisfaction among men. In contrast, among women, the explained variance reached 6.1% for competence satisfaction, 0.5% for competence frustration, and 51.7% for life satisfaction. The structural relationships between teaching efficacy, competence satisfaction, competence frustration, and life satisfaction, along with indirect effects, are presented in Figure 1 and Table 3, with results reported separately for male and female groups.

The multigroup SEM revealed distinct predictive patterns between male and female pre-service PE teachers in the prediction of teaching efficacy to life satisfaction, highlighting the mediating role of competence need-experiences. For male participants, teaching efficacy significantly and positively predicted competence satisfaction and negatively predicted competence frustration. In turn, only competence satisfaction was a significant predictor of life satisfaction, while competence frustration showed no significant effect. A significant indirect effect was found from teaching efficacy to life satisfaction through the mediating variables. In the female group, teaching efficacy also positively predicted competence satisfaction and negatively predicted competence frustration. Among women, only competence satisfaction significantly predicted life satisfaction, with frustration showing no effect. The indirect pathway from teaching efficacy to life satisfaction was also significant.

## 4. Discussion

The present study had two main objectives. The first was to examine gender differences in pre-service teachers’ perceptions of their teaching efficacy, competence satisfaction, competence frustration, and life satisfaction. The second objective was to analyze whether teacher’s sense of efficacy exerts differential effects on competence frustration, competence satisfaction, and life satisfaction among male and female pre-service PE teachers. The main findings of the study revealed that (i) gender differences emerged only in perceptions of competence satisfaction, with teaching efficacy being associated differently for males and females; (ii) teaching efficacy was similarly related to life satisfaction through competence satisfaction in both groups, although the indirect effect was slightly stronger among females; and (iii) consistent with H1, a direct association between teaching efficacy and life satisfaction emerged among women, whereas this path was not significant among men once competence-related mediators were included.

With regard to the first objective, the results of the present study identified gender differences only in competence satisfaction, with male pre-service PE teachers reporting significantly higher levels than their female counterparts. These findings contrast with previous research suggesting that perceptions of the need for competence are generally similar across genders [29]. This discrepancy may be partly explained by the specific context of PE teacher training, where performance-based tasks and physical competence may be perceived or evaluated differently by male and female students. However, due to the limited number of studies focused specifically on pre-service PE teachers, further research is required to clarify the origins of these gender differences. In particular, empirical studies could provide valuable insights into the underlying factors, such as cultural expectations, pedagogical experiences, or self-perceptions of skill, that shape students’ interpretations of their own competence during teacher education. Understanding these gendered dynamics is crucial for developing more inclusive and equitable approaches to supporting psychological need satisfaction in initial teacher training programs.

Regarding the second objective, the results revealed that pre-service teachers’ perceived teaching efficacy had a direct and positive effect on life satisfaction among female pre-service teachers (supporting H1) while this direct effect was not significant among men when competence-related mediators were included. These findings are consistent with previous studies [7], as well as with the theoretical assumptions of SCT [6]. Specifically, according to SCT, a teacher’s sense of efficacy influences how individuals evaluate their ability to perform professional tasks, which in turn shapes their expectations for future success and contributes to a more positive sense of well-being, including greater life satisfaction. This effect may be explained by Bandura’s [6] assertion that individuals function as intentional agents, capable of influencing their own development and outcomes through their beliefs and actions. In this sense, the cognitive representation of one’s ability to perform competently in the teaching profession becomes a powerful motivational resource. It not only guides behavior but also reinforces perceptions of effectiveness and fulfillment, ultimately enhancing psychological well-being and life satisfaction among pre-service teachers.

Consistent with the hypothesized model, the results of this study revealed a significant association between teaching efficacy and the satisfaction of the basic psychological need for competence, which in turn was positively related to life satisfaction (supporting H2 in both groups). These findings are supported by previous research, such as that of Raven and Kleinert [14] and Kassis et al. [15], which emphasize that perceived competence satisfaction contributes to enhanced life satisfaction. In line with prior evidence, competence satisfaction is a prospective predictor of students’ well-being and life satisfaction in educational contexts [14,15,20,50]. From a Self-Determination Theory perspective, competence-affirming experiences foster internalization and goal-directed engagement, which, in turn, contribute to higher subjective well-being [50]. Similarly, the present findings align with existing literature that identifies competence satisfaction as a core need capable of driving positive well-being outcomes in university settings [50]. This suggests that pre-service teachers’ belief in their ability to organize and execute the actions required for effective teaching, combined with the intrinsic satisfaction derived from meeting the academic and professional challenges of teacher training, fosters a stronger sense of subjective well-being. One possible explanation is that pre-service teachers perceive teaching as a profession with high cognitive and procedural demands (e.g., preparation for competitive public examinations) [51]. In such cases, even if students believe they are capable of performing the role of a teacher, if the actual requirements lack sufficient complexity to challenge and engage their sense of competence, they may not receive the type of positive feedback necessary to reinforce teaching efficacy beliefs [12]. Moreover, studies such as that by Vasconcellos et al. [52] highlight the central role of competence satisfaction in the development of self-regulated motivational processes and in promoting subjective well-being, particularly in physical education students. By contrast, competence frustration did not significantly mediate the relationship between teaching efficacy and life satisfaction in either group (H3 not supported).

Finally, regarding the gender-based analysis, and in line with the present study, Erbaş et al. [3] found that perceptions of teaching efficacy among pre-service PE teachers varied by gender. First, competence satisfaction played a significant mediating role between teaching efficacy and life satisfaction for both male and female participants (supporting H2 in both groups). However, although the total effects were similar, the predictive pathways differed by gender. Female students showed stronger associations between competence satisfaction and life satisfaction, whereas male students exhibited stronger links between teaching efficacy and competence satisfaction [53]. According to Erbaş et al. [3], this may be due to the fact that male students tend to internalize their perceptions of future teaching performance more deeply during their training process, which in turn enhances their sense of competence [54]. Second, the direct association between teaching efficacy and life satisfaction was evident among women but not among men (H1). A plausible explanation for this could lie in the influence of a more developed professional identity, which may shape how teaching efficacy contributes to perceptions of well-being [55]. Although critical perspectives on teacher identity have gained traction in the study of pre-service PE teachers [56], this construct was not examined in the present research. Third, competence frustration did not act as a mediator for either gender (H3 not supported), reinforcing the primacy of competence satisfaction over frustration in explaining life satisfaction within this cohort.

### 4.1. Limitations and Future Prospects

Despite the above findings, the present research has several limitations that should be acknowledged. First, although the model integrated SDT and SCT, causal inferences cannot be made due to the cross-sectional nature of the study. Future research should adopt longitudinal designs to examine how teaching efficacy influences well-being outcomes before and after the teacher training process. Second, the sample used in this study was homogeneous and selected by convenience, limited to a specific teaching specialization (i.e., Physical Education), a particular stage of career development (i.e., pre-service teachers), and a specific type of training (i.e., MAES) [57]. Therefore, future studies should explore whether similar patterns are found among pre-service teachers from other subject areas. Third, we did not collect detailed indicators of practicum engagement (e.g., course attendance, presence in placement schools, teaching hours, diversity of groups taught, mentoring/feedback frequency), which may shape efficacy beliefs, competence experiences, and sources of satisfaction/dissatisfaction. Future work should include these indicators and, ideally, combine self-reports with mentor ratings/observations in multi-method and longitudinal designs. Finally, the use of non-probability sampling suggests that the findings should be interpreted with caution. Further research is needed to investigate whether perceptions of competence influence the relationship between teaching efficacy and life satisfaction differently for male and female in-service teachers, and whether these patterns vary according to their years of teaching experience.

### 4.2. Practical Implications for Pre-Service Teacher Education

In light of the findings from the present study, several educational implications can be proposed, particularly for teacher educators concerned with supporting the subjective well-being of their students [58]. The results indicate that, regardless of gender, competence satisfaction acts as a mediating variable between teaching efficacy and life satisfaction among pre-service PE teachers. In this regard, teacher educators should foster a learning climate that promotes the satisfaction of the psychological need for competence [50,52]. This could involve conducting prior assessments of the content to be taught (e.g., evaluation of pedagogical models and strategies used in practice), setting clear goals and challenges for students (e.g., designing a learning situation focused on a specific PE content), creating tasks with varying degrees of difficulty (e.g., a didactic sequence for the progression of a gymnastic skill), and providing feedback and guidance based on the learning outcomes achieved through those challenges (e.g., offering prescriptive and formative feedback on classroom tasks) [59,60]. Considering the gender differences found in the relationship between teaching efficacy and life satisfaction, it is clear that pre-service PE teachers need to experience competence satisfaction. However, the perception of high teaching efficacy appears to be especially relevant in predicting life satisfaction among female students [61]. Therefore, exploring the factors that enhance teachers’ sense of efficacy could be particularly beneficial for female pre-service PE teachers.

## 5. Conclusions

Although gender differences were found only in competence satisfaction, the effects of teaching efficacy differed between male and female participants. Overall, the results of this study suggest that, while teaching efficacy was associated with life satisfaction through competence satisfaction in both groups, the relationship between teaching efficacy and competence satisfaction was stronger among male pre-service teachers. Conversely, the findings highlight the importance of the direct relationship between teaching efficacy and life satisfaction among female pre-service PE teachers, underscoring the relevance of perceived teaching efficacy as a key predictor of subjective well-being in this group.

## Figures and Tables

**Table 1:** Descriptive statistics and correlations in pre-service teachers.

	Women (*n* = 171)		Men (*n* = 197)		Correlations			
Variables	M	SD	M	SD	1	2	3	4
1. SEE	7.51	1.01	7.54	0.89	-	0.21 **	−0.02	0.092
2. Competence Satisfaction	3.89	0.75	4.04	0.62	0.38 **	-	0.53 **	0.59 **
3. Competence Frustration	2.22	0.89	2.27	0.89	−0.27 **	−0.45 **	-	−0.44 **
4. Life Satisfaction	3.79	0.84	3.85	0.77	0.18 **	0.37 **	−0.23 **	-

Note: SEE = sense of teaching efficacy; SD = standard deviation; numbers above the diagonal show the correlations for female pre-service teachers. Numbers below the diagonal show the correlations for male PE pre-service teachers. ** *p* < 0.01.

**Table 2:** Mean differences between pre-service teachers.

	Women (*n* = 171)		Men (*n* = 197)		Mean Difference			
Variables	M	SD	M	SD	Dif	t(gl)	*p*-Value	d
1. SEE	7.51	1.01	7.54	0.89	−0.32	−0.26(366)	0.396	0.30
2. Competence Satisfaction	3.89	0.75	4.04	0.62	−0.14	−2.07(366) ^a^	0.20	0.08
3. Competence Frustration	2.22	0.89	2.27	0.89	−0.04	−0.52(366)	0.301	0.07
4. Life Satisfaction	3.79	0.84	3.85	0.77	−0.05	−0.70(366)	0.240	0.15

Note: SEE = sense of teaching efficacy; SD = standard deviation; Dif = mean differences; d = Cohen’s d; ^a^ = Levene’s test is significant, the variances are not equal.

**Table 3:** Multigroup SEM results by gender (women compared to men).

Independent Variable		Dependent Variable	Mediator	β	SE	95% CI	
						Inf	Sup
Women							
Direct effects							
	SEE	Competence Satisfaction		0.25 **	0.10	0.03	0.45
	Competence Satisfaction	Life Satisfaction		0.38 **	0.13	0.25	0.77
Indirect effects							
	SEE	Life Satisfaction	Competence Satisfaction	0.13 **	0.06	0.01	0.25
Men							
Direct effects							
	SEE	Competence Satisfaction		0.53 *	0.08	0.37	0.68
	SEE	Competence Frustration		−0.24 **	0.07	0.40	0.09
	SEE	Life Satisfaction		0.12 **	0.09	−0.08	0.29
	Competence Satisfaction	Life Satisfaction		0.33 **	0.06	0.31	0.47
Indirect effects							
	SEE	Life Satisfaction	Competence Satisfaction	0.19 **	0.07	0.06	0.30

Note. β = estimation of standardized parameters; SE = standard error; 95% CI = 95% confidence interval; Inf = lower limit; Sup = upper limit; ** *p* < 0.01; * *p* < 0.05.

## Data Availability

Data is contained within the article.

## Abbreviations

The following abbreviations are used in this manuscript:

SCT	Social Cognitive Theory
PE	Physical Education
SDT	Self-Determination Theory
MAES	Master’s in Secondary Education Teaching
CFI	Comparative Fit Index
TLI	Tucker–Lewis Index
RMSEA	Root Mean Square Error of Approximation
SRMR	Standardized Root Mean Square Residual
